# Adaptive Neural Network-Based Tracking Control for a Single-Link Flexible Manipulator Under State Constraints

**DOI:** 10.3390/s26123722

**Published:** 2026-06-11

**Authors:** Enrui Liu, Wuxing Lai, Songyi Dian

**Affiliations:** 1 Pittsburgh Institute, Sichuan University, Chengdu 610207, China; 2023141520014@stu.scu.edu.cn; 2 College of Electrical Engineering, Sichuan University, Chengdu 610207, China; laiwuxing0830@163.com

**Keywords:** single-link flexible manipulator, fractional order calculus, barrier Lyapunov function, radial basis function neural network

## Abstract

Flexible manipulators have attracted increasing attention due to their lightweight structure, high flexibility, and energy efficiency, for which they are suitable for delicate and high-precision tasks. However, their control remains a problem because of strong nonlinearities and uncertainties in the system. Based on the trajectory tracking control problem of the single-link flexible manipulator (SLFM) system, this paper proposes a fractional order adaptive neural network control scheme for SLFM under symmetric time-varying full-state constraints. Firstly, a fractional-order dynamic model is established to better capture the inherent memory and nonlinear characteristics of the SLFM. Secondly, an adaptive radial basis function (RBF) neural network-based control scheme is developed within a backstepping framework, and a symmetric time-varying barrier Lyapunov function (BLF) is incorporated to guarantee that all system states remain within predefined bounds. In addition, command filters are introduced to avoid the “explosion of complexity” caused by backstepping. Next, theoretical analysis based on Lyapunov stability theory is provided to demonstrate that all signals in the closed-loop system are bounded, while the tracking error converges to a small neighborhood of zero. Finally, the proposed method is applied as an SLFM: the simulation results show that the presented controller has excellent control performance, the tracking error is less than 0.02 rad, and the tip polarization angle of the system does not exceed 0.045 rad. Additionally, the comparison with the recent DSC and SMC methods also shows that the designed controller behaves with less tracking error, which in return validates the effectiveness and superiority of the proposed control strategy.

## 1. Introduction

Robots are playing an increasingly important role in a wide range of fields, including intelligent manufacturing, surgical assistance, and aerospace exploration. In particular, robotic manipulators serve as the core components of robotic systems, as they directly interact with the physical environment under the guidance of programmed control algorithms. This enables them to perform skill-intensive, hazardous, repetitive, and labor-intensive tasks with high precision and efficiency [1]. Consequently, the design and control of robotic manipulators have attracted sustained attention in both academia and the industry.

Among various types of manipulators, single-link flexible manipulators (SLFMs) have received significant attention in recent years. Compared with traditional rigid manipulators, SLFMs exhibit superior performance in delicate and high-precision tasks due to their inherent structural flexibility. Moreover, they possess effectively infinite degrees of freedom, which greatly enhance their adaptability in complex environments [2,3]. These advantages primarily stem from their intrinsic properties, such as lightweight structure, low energy consumption, and strong dexterity [4,5,6,7,8]. As a result, SLFMs are particularly suitable for applications requiring high flexibility and safety.

However, the inherent flexibility of SLFMs also introduces several challenging issues. Specifically, structural flexibility leads to unwanted vibrations and dynamic uncertainties, which significantly complicate the control design [9]. In practical applications, SLFMs are often subject to various uncertainties, including external disturbances, unmodeled dynamics, and parameter variations, all of which degrade the accuracy of the system model [10,11]. Furthermore, the system dynamics are highly nonlinear and strongly coupled, making it difficult to achieve high-performance control [12]. In addition, for safety and operational requirements, system states such as joint angles and tip deflections are usually required to strictly satisfy predefined constraints. The presence of such state constraints further increases the complexity of controller design and stability analysis.

To address the aforementioned challenges, extensive research efforts have been devoted to both the modeling and control design of SLFMs. From the perspective of system modeling, several classical approaches have been widely adopted. For instance, Zhang et al. [13] established a flexible two-link manipulator model based on the Hamiltonian principle. Scaglioni et al. [14] developed a three-dimensional flexible manipulator model using the Newton–Euler formulation. Wang and Jiang [15] employed the Lagrange method combined with the assumed modal method to derive the dynamics of flexible link–flexible joint manipulators. These methods represent the most commonly used techniques for modeling SLFMs. Nevertheless, most existing studies rely on integer-order calculus (IOC) for system modeling. Considering that SLFMs exhibit inherent memory effects and complex nonlinear behaviors, fractional-order modeling provides a more accurate and effective framework. Fractional-order derivatives are particularly suitable for describing systems with memory, hereditary properties and long-term dependencies, thereby offering improved modeling fidelity compared with traditional integer-order approaches [16,17].

In terms of controller design, a variety of advanced control strategies have been proposed. Among them, adaptive control and neural network-based control have gained considerable attention due to their ability to handle uncertainties and nonlinearities. For example, Zhao et al. [18] developed an adaptive control scheme to suppress disturbances and elastic oscillations. Many other researchers adopt the adaptive fuzzy control scheme for flexible robotic systems specifically [19,20]. Shang et al. [21] proposed a sliding mode control approach combined with radial basis function (RBF) neural networks to approximate system uncertainties. Zhang et al. [22] incorporated RBF neural networks into cooperative trajectory tracking control to compensate for unknown and unmodeled dynamics. Gao et al. utilized an adaptive RBF neural network-based controller for a two-link flexible robotic manipulator to improve robustness and handle system uncertainties [23]. Although these methods effectively mitigate the influence of uncertainties and disturbances, most existing works do not explicitly consider full-state time-varying constraints during controller design, which may limit their applicability in safety-critical scenarios. To address this issue, the barrier Lyapunov function (BLF) can be utilized, which is recognized as a powerful approach to deal with a constrained nonlinear system [24,25]. By transforming state constraints into state error signals, this indirect method guarantees that the constraints are not violated once the BLF chosen is kept bounded.

Motivated by the above observations, this paper proposes an adaptive neural network control scheme for fractional-order SLFMs with full-state constraints. Specifically, the contributions are as follows:

- A fractional-order dynamic model is established to accurately describe the system behavior;
- An adaptive RBF neural network-based controller is developed to approximate unknown nonlinear dynamics, while BLF is incorporated to ensure that all system states satisfy prescribed time-varying constraints.

Furthermore, a rigorous stability analysis based on Lyapunov theory is conducted to guarantee the boundedness of all closed-loop signals. Finally, simulation results are presented to demonstrate the effectiveness and superiority of the proposed control strategy.

The remainder of this paper is organized as follows. Section 2 presents the system model and necessary preliminaries. Section 3 details the controller design. Section 4 provides the stability analysis. Section 5 gives simulation results, followed by conclusions in Section 6.

## 2. Problem Statement and Preliminaries

In this chapter, the fractional order derivative dynamic model of the single-link flexible manipulator will be presented, and some preliminary knowledge and necessary lemmas are listed to facilitate the subsequent controller design and stability analysis.

### 2.1. Problem Formulation

Before the construction of the dynamic model, make the following assumptions first:

**Assumption** **1.** *The rotating axis of the motor rotor is co-axial with the manipulator’s rotating axis.*

**Assumption** **2.** *The influence of the motor’s electrical dynamics can be ignored compared to its mechanical dynamics.*

**Assumption** **3.** *The rotational kinetic energy of the rotor is determined by manipulator’s velocity as the rotor’s rotational inertia is much smaller than the manipulator’s rotational inertia.*

**Assumption** **4.** *The influences of shearing deformation and the gravity of the manipulator are ignored under the premise that SLFM undergoes small deformation.*

**Figure 1:**
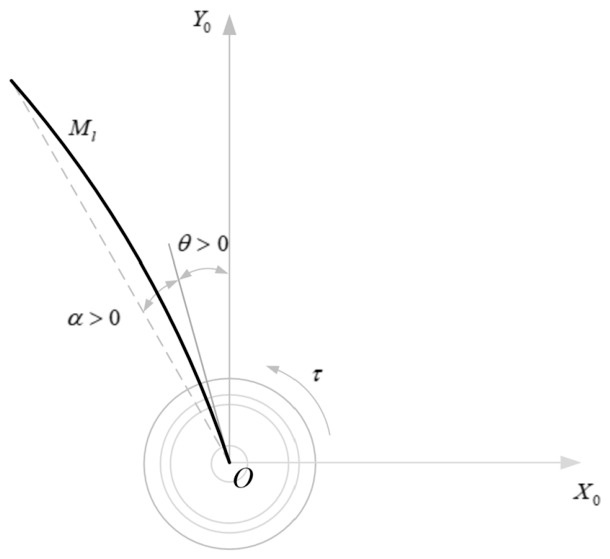
Schematic of the SLFM system.

The schematic of the SLFM system is shown in Figure 1. $X_{0}OY_{0}$ is the fixed coordinate system, $M_{l}$ is the mass of the manipulator, τ is the control torque provided by the output voltage, θ is the rotation angle of the manipulator, and α represents the bending deformation at the tip.

The SLFM system is a nonlinear mechanical system with incomplete constraints. Lagrange energy functions are used to model it, and the total energy of the system is given by

(1) L=T−V,

where T and V represent the kinetic and potential energy of the system, respectively.

Only horizontal movements for SLFM are studied in this article, and the total kinetic energy is given by

(2) $$T=\frac{1}{2}J_{eg}{\dot{\theta }}^{2}+\frac{1}{2}J_{l}{\left(\dot{\theta }+\dot{\alpha }\right)}^{2}$$

Here, $J_{eq}$ is the motor’s rotational inertia, and $J_{l}$ is the link’s rotational inertia, both with respect to the rotational axis. The total potential energy of the system is

(3) $$V=\frac{1}{2}K_{s}\alpha^{2},$$

where $K_{s}$ is the flexible manipulator’s link rigidity. Combine (1)–(3) to get

(4) $$L=\frac{1}{2}J_{eq}{\dot{\theta }}^{2}+\frac{1}{2}J_{l}(\dot{\theta }+\dot{\alpha }{)}^{2}-\frac{1}{2}K_{s}\alpha^{2}$$

According to Lagrange equations of the second kind:

(5) $$\left\{\begin{array}{l} \frac{\partial^{2}L}{\partial t\partial \dot{\theta }}-\frac{\partial L}{\partial \theta }=Q_{1}=\tau -B_{eq}\dot{\theta }\\ \frac{\partial^{2}L}{\partial t\partial \dot{\alpha }}-\frac{\partial L}{\partial \alpha }=Q_{2}=-B_{l}\dot{\alpha } \end{array}\right.$$

$Q_{1}$ and $Q_{2}$ are generalized forces, and $B_{eq}$ is the viscous damping coefficient of the servo system. $B_{l}$ is the viscous damping coefficient related to the friction on the link, which is set to be 0 in this article for simplification. Substituting (4) into (5) will yield:

(6) $$\left\{\begin{array}{l} \ddot{\theta }=-\left(B_{eq}/J_{eq}\right)\dot{\theta }+K_{s}\alpha +\left(1/J_{eq}\right)\tau \\ \ddot{\alpha }=\left(B_{eq}/J_{eq}\right)\dot{\theta }-K_{s}\left(\left(J_{l}+J_{eq}\right)/J_{l}J_{eq}\right)\alpha -\left(1/J_{eq}\right)\tau \\ y=\alpha +\theta \end{array}\right.$$

Rewrite the equation set in state space representation:

(7) $$\left\{\begin{array}{c} \dot{\eta_{1}}=\eta_{3}\\ \dot{\eta_{2}}=\eta_{4}\\ \dot{\eta_{3}}=\omega_{2}\eta_{2}-\omega_{1}\eta_{3}+\omega_{3}u(t)\\ \dot{\eta_{4}}=-\omega_{4}\eta_{2}+\omega_{1}\eta_{3}-\omega_{3}u(t)\\ y=\eta_{1}+\eta_{2} \end{array}\right.$$

Here, we define $\eta^{T}=[\theta ,\alpha ,\dot{\theta },\dot{\alpha }]$, $u\left(t\right)=\tau$, $\omega_{1}=\frac{B}{J_{eq}}$, $\omega_{2}=\frac{K}{J_{eq}}$, $\omega_{3}=\frac{1}{J_{eq}}$, and $\omega_{4}=\;\frac{K(J_{l}+J_{eq})}{J_{l}J_{eq}}$. Due to the fact that SLFM is an underactuated system, introduce the following global coordinate transformation to decouple the system into two second-order subsystems:

(8) $$\left\{\begin{array}{c} x_{1}=\eta_{1}+\eta_{2}\\ x_{2}=\eta_{3}+\eta_{4}\\ x_{3}=\eta_{2}\\ x_{4}=\eta_{4} \end{array}\right.$$

Then, substitute (7) into (8) and apply fractional calculus to handle the nonlinearities and memory effects of the SLFM system. Based on the fractional-order definition given in Section 2.2, the system dynamics can be described as follows:

(9) Dtq x1=x2+f1(x1)0C+d1(t)Dtq x2=x3+f2(x3)0C+d2(t)Dtq x3=x4+f3(x3)0C+d3(t)Dtq x4=−ω3u(t)+f4(x2,x3,x4)0C+d4(t)y=x1

In the following equations, $f_{1}(x_{1})=f_{3}(x_{3})=0$, $f_{2}(x_{3})=(\omega_{2}-\omega_{4}-1)x_{3}$, and $f_{4}(x)=\omega_{1}(x_{2}-x_{4})-\omega_{4}x_{3}$. $d_{i}(t)(i=1,\;2,\;3,\;4)$ are unknown disturbances introduced to the state space equations, and there exists an upper bound for each disturbance: $d_{i}(t)\leq \Lambda_{i}^{*}$. u(t)∈R is the input of the system. $x_{1},x_{2},x_{3},x_{4}$ represent the state variables for the system, and y is the output of the system. Define an open set as $\Omega_{x_{i}}:=\{x_{i}(t)\in R:|x_{i}(t)|<k_{c_{i}},k_{c_{i}}>0\}$, i=1, 2, 3, 4, ∀t>0. $k_{c_{i}}$ is a positive constant number and every state variable is constrained in $\Omega_{x_{i}}$.

The objective of this paper is to develop an adaptive neural network control scheme for the single-link flexible manipulator system described above, which is subject to multiple time-variant constraints as well. Specifically, the controller is designed to achieve the following objectives:

(1) The system output tracks the desired trajectory with high accuracy, i.e., the tracking error converges to a small neighborhood of zero.

(2) All signals in the closed-loop system remain bounded.

(3) The full-state constraints are strictly satisfied, ensuring that all state variables remain within predefined time-varying bounds.

(4) The effects of unknown nonlinear dynamics and external disturbances are effectively compensated.

### 2.2. Preliminary Knowledge and Lemmas

To realize the aforementioned objectives, the following preliminary knowledge and lemmas are worth mentioning.

#### 2.2.1. Fractional Calculus

There are different forms of definitions for fractional calculus, and the most commonly seen forms are the R-L form, Caputo form, and G-L form. For this article, the Caputo form fractional order derivative is used, and its definition is provided below [26]:

(10) Dt0Ctqf(t)=1Γ(n−q)∫t0tf(n)(s)(t−s)q−n+1ds

In the definition, $t\geq t_{0}$ and n=[q]. When n−1<q≤n, introduce the Mittag-Leffler function as follows [27]:

(11) $$\phi_{q}(t)=\sum_{k=0}^{\infty }\frac{t^{k}}{\Gamma \left(kq+1\right)},q>0$$

For $t_{0}=0$ and 0<q<1, it can be obtained that [28]:

(12) D0Ctqf(t)=1Γ(1−q)∫0tf′(s)(t−s)qds

**Lemma** **1** ([29])**.** *Let* $x(t)=[x_{1}(t),x_{2}(t),\ldots ,x_{n}(t){]}^{T}\in R^{n}$ *be a continuous and differentiable vector function. If* $t\geq t_{0},\;q\in (0,\;1)$ *, then:*

Dt0CtqxT(t)Px(t)≤xT(t)PDt0Ctqx(t)+Dt0CtqxT(t)Px(t),

*where* $P\in R^{n\times n}$ *is a symmetric and positive definite matrix.*

**Lemma** **2** ([30])**.** *If* $h_{1}$ *and *$h_{2}$* are both smooth functions, and *$\frac{\partial^{2}h_{1}h_{2}}{\partial h_{2}^{2}}\geq 0$*, then *∀t≥0, ∃D0Ctqh1h2≤∂h1h2∂h2·D0Ctqh2*, where *q∈(0, 1].

**Lemma** **3** ([31])**.** $\forall k_{b_{0}}>0$*, if *$|\varsigma (t)|\leq k_{b_{0}}$*, then *$\ln\frac{k_{b_{0}}^{2}}{k_{b_{0}}^{2}-\varsigma^{2}(t)}\leq \frac{\varsigma^{2}(t)}{k_{b_{0}}^{2}-\varsigma^{2}(t)}$.

**Lemma** **4** ([32])**.** *If the Caputo fractional order derivative of continuous function* V(t): [0, +∞)→R *satisfies that *Dt0CtqV(t)≤−aV(a)+b, t≥t0≥0, a>0, b>0*, then *$V(t)\leq \left(V(t_{0})-\frac{b}{a}\right)\phi_{q}\left(-a(t-t_{0}{)}^{q}\right)+\frac{b}{a},t\geq t_{0}\geq 0$.

#### 2.2.2. Radial Basis Function Neural Network

The radial basis function neural network is a three-layer feedforward neural network. In the field of nonlinear adaptive control, it is frequently used to deal with unknown smooth nonlinear functions, as it can approximate a function well.

For a continuous function $f(x):R^{n}\to R$ and any ε>0, given a compact set $\Xi_{n}\subset R^{n}$, there exist radial basis function neural networks (RBF NNs) such that:

$$|f(x)-W^{T}\Phi (x)|\leq \varepsilon$$

Here, $W=[w_{1},\cdots ,w_{\iota }{]}^{T}$ is the weight vector, ι>1 is the number of network nodes, and $x\in R^{n}$ is the input. $\Phi (x)=[\phi_{1}(x),\cdots ,\phi_{\iota }(x){]}^{T}$ is the RBF vector, where the Gaussian function is in the following form:

$$\phi_{i}(x)=\exp\left(\frac{-(x-\mu_{i}{)}^{T}(x-\mu_{i})}{\eta_{i}^{2}}\right),$$

where i=1, 2, ..., ι, $\mu_{i}$ is the center of the neuron, and $\eta_{i}$ is the width of Gaussian function.

**Figure 2:**
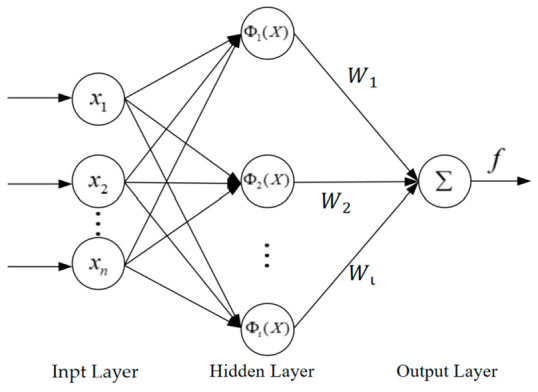
The structure for RBF neural network.

The structure for the RBF neural network is shown in Figure 2 below:

The unknown functions in the system can be approximated as

$$f_{i}(x)={W_{i}^{*}}^{T}\Phi_{i}(x)+\varepsilon_{i}(x)$$

$W_{i}^{*}$ is the ideal weight and is defined as

$$W_{i}^{*}=\arg\underset{W_{i}\in R^{\iota }}{min}\left\{\underset{x_{i}\in \Xi_{i}}{sup}\left|f_{i}(x)-{\hat{W}}_{i}^{T}\Phi_{i}(x)\right|\right\}$$

${\hat{W}}_{i}$ is the estimation of $W_{i}^{*}$. Meanwhile, $0<\Phi_{i}^{T}(\cdot )\Phi_{i}(\cdot )\leq \iota_{i}$, where $\iota_{i}$ is the number of neurons.

## 3. Controller Design

Next, design the controller for the SLFM dynamic system. The following assumptions are made for this chapter:

**Assumption** **5** ([33])**.** *Assume the reference signal* $y_{d}$ *and its fractional order derivatives *D0Ctqyd *and *D0Ctq(D0Ctqyd) *are continuous and bounded, which means *$|y_{d}|\leq A_{1}<k_{c_{i}}$*, *D0Ctqyd≤Y1 *and *D0Ctq(D0Ctqyd)≤Z1*, where *$A_{1},\;Y_{1},\;Z_{1}>0$.

**Assumption** **6** ([34])**.** *Unknown nonlinear function *$f_{i}(x)$ *satisfies local Lipschitz condition, which means there exists a known constant* $\zeta_{i}>0$ *such that for any* $x,y\in R^{n}$ *,* $\left|f_{i}(x)-f_{i}(y)|\leq \zeta_{i}\|x-y\right\|$ *is always satisfied. Here,* ‖x‖ *represents the 2-norm of vector **x**.*

In this chapter, the adaptive neural network control algorithm combined with the fractional order command filter is used. Meanwhile, adaptive parameters are applied to effectively estimate all of the disturbances in the system, increasing the robustness of the system. The command filter [35] is defined as follows:

(13) Dtq0Cφi,1=βj−1φi,2Dtt0Cφi,2=−2ξβj−1φi,2−βj−1φi,1−zj−1

In the equations, $\varphi_{i,1}=x_{i,d}$ is the output of the command filter, and $z_{j-1}$ is the input. ξ∈(0, 1) and $x_{i,d}(0)=z_{j-1}(0),\;\varphi_{i,2}(0)=0$ when j = 2, 3, 4. Conduct the following coordinate transformation:

(14) $$\left\{\begin{array}{l} S_{1}=y-y_{d}\\ S_{i}=x_{i}-x_{i,d}\;i=2,\;3,\;4\\ v_{i}=S_{i}-r_{i} \end{array}\right.$$

$S_{i},\;r_{i},\;v_{i}$ represent the tracking error, compensated signal, and compensated tracking error signal, respectively.

Based on the approximation characteristic of RBF NNs, the unknown functions in the system can be approximated as

(15) $$f_{i}(x)={W_{i}^{*}}^{T}\Phi_{i}(x)+\varepsilon_{i}(x)$$

$W_{i}^{*}$ is the ideal weight and is defined as

(16) $$W_{i}^{*}=\arg\underset{W_{i}\in R^{\iota }}{min}\left\{\underset{x_{i}\in \Xi_{i}}{sup}\left|f_{i}(x)-{\hat{W}}_{i}^{T}\Phi_{i}(x)\right|\right\}$$

${\hat{W}}_{i}$ is the estimation of $W_{i}^{*}$.

The following part will present the design process of the controller in four steps.

**Step 1:**

The fractional order derivative of the compensated tracking error signal $v_{1}$ is calculated as:

(17) D0Ctqv1=D0Ctqy−D0Ctqyd−D0Ctqr1=v2+r2+x2,d+W1*TΦ1(x)+ϵ1(x)+W^1TΦ1(x1)+W~1TΦ1(x1)−W1*TΦ1(x1)+d1(t)−Dtq0Cyd−D0Ctqr1

The Lyapunov function is designed as:

(18) $$V_{1}=\frac{1}{2}\ln\frac{k_{b_{1}}^{2}}{k_{b_{1}}^{2}-v_{1}^{2}}+\frac{1}{2\Gamma_{1}}{\tilde{W}}_{1}^{T}{\tilde{W}}_{1}+\frac{1}{2\gamma_{1}}{\tilde{\Lambda }}_{1}^{2}$$

In the function, $\Gamma_{1},\;\gamma_{1}>0,\;\left|v_{1}\right|<k_{b_{1}}=k_{c_{1}}-\Lambda_{1}$. It is worth noting that the barrier Lyapunov function remains a positive definite if $\left|v_{1}\right|<k_{b1}$, which helps to restrict the state of $x_{1}$ and keeps the system stable. It could then be derived later that $|x_{1}|\leq |v_{1}|+|y_{d}|+|r_{1}|<k_{b_{1}}+\Lambda_{1}$.

According to Lemmas 1–3 and (18), it can be derived that:

(19) D0CtqV1≤v1kb12−v12Dtq0Cv1+1Γ1W1T~D0CtqW1~+1γ1Λ1~D0CtqΛ1~=v1kb12−v12(v2+r2+x2,d+W1*TΦ1(x)+ϵ1(x)+d1(t)−D0Ctqyd−D0Ctqr1)−1Γ1W~1TD0CtqW1−1γ1Λ1~D0CtqΛ1

According to Young’s inequality:

(20) $$\frac{v_{1}}{{k_{b_{1}}}^{2}-v_{1}^{2}}\varepsilon_{1}(x)\leq \frac{v_{1}^{2}}{2({k_{b_{1}}}^{2}-v_{1}^{2}{)}^{2}}+\frac{{\bar{\varepsilon }}_{1}^{2}}{2}$$

(21) $$\frac{v_{1}}{k_{b_{1}}^{2}-v_{1}^{2}}W_{1}^{*T}\left(\Phi_{1}(x)-\Phi_{1}(x_{1})\right)\leq \frac{\|W_{1}^{*}{\|}^{2}v_{1}^{2}}{(k_{b_{1}}^{2}-v_{1}^{2}{)}^{2}}+N,\;N>\iota_{1}$$

Substitute (20) and (21) into (19):

(22) D0CtqV1≤v1kb12−v12(v2+r2+x2,d−z1+z1 + W^1TΦ1(x1)+W~1TΦ1(x1)+d1(t)−Dtq0Cyd−D0Ctqr1) +v122(kb12−v12)2+‖W1*‖2v12(kb12−v12)2−1Γ1W~1TD0CtqW1−1γ1Λ1~D0CtqΛ1+N+ϵ¯122

Select the pseudo control law $z_{1}\;$and compensated signal $r_{1}$ as follows:

(23)  z1=−c1S1−S12(kb12−v12)−Λ1tanhv1ϖ−W^1TΦ1(x1)+D0CtqydD0Ctqr1=−c1r1−r12(kb12−v12)+r2+x2,d−z1

The adaptive law is designed in the following form:

(24) D0CtqΛ1=γ1v1kb12−v12tanhv1ϖ−τ1Λ1D0CtqW1=Γ1v1kb12−v12Φ1x1−σ1W1

Substitute (23) and (24) into (22), and the final form of the fractional order derivative of the barrier Lyapunov function $V_{1}$ is shown below:

(25)  D0CtqV1≤1kb12−v12−c1−‖W1*‖2v12+v1v2+τ1γ1Λ~1Λ1+σ1Γ1W~1TW1+M1,

where $M_{1}=N+{\bar{\epsilon }}_{1}^{2}/2+0.2785\Lambda_{1}^{*}\varpi /(k_{b_{1}}^{2}-\nu_{1}^{2})$.

**Step i, i = 2, 3**: Using similar approaches as in **Step 1**, the fractional order derivative of the compensated signal $v_{i}$ is calculated to be:

(26) D0Ctqvi=D0Ctqxi−D0Ctqxi,d−D0Ctqri=vi+1+ri+1+xi+1,d+Wi*TΦi(x)+εi(x)+W^iTΦi(xi)+W~iTΦi(xi)−Wi*TΦi(xi)+di(t)−D0Ctqxi,d−D0Ctqri

The Lyapunov function is selected as:

(27) $$V_{i}=V_{i-1}+\frac{1}{2}\ln\frac{k_{b_{i}}^{2}}{k_{b_{i}}^{2}-v_{i}^{2}}+\frac{1}{2\Gamma_{i}}{\tilde{W}}_{i}^{T}{\tilde{W}}_{i}+\frac{1}{2\gamma_{i}}{\tilde{\Lambda }}_{i}^{2}$$

$\Gamma_{i},\;\gamma_{i}>0,\left|v_{i}\right|<k_{b_{i}}=k_{c_{i}}-\Lambda_{i}$. According to Lemmas 1–3, the fractional order derivative of the barrier Lyapunov function is:

(28)  D0CtqVi≤ D0CtqVi−1+vikbi2−vi2(vi+1+ri+1+xi+1,d−zi+zi +Wi*TΦi(x)+ ϵi(x)+di(t)−D0Ctqxi,d−D0Ctqri)−1ΓiW~iTD0CtqWi−1γiΛ~iD0CtqΛi

According to Young’s inequality:

(29) $$\frac{v_{i}}{{k_{b_{i}}}^{2}-v_{i}^{2}}\varepsilon_{i}(x)\leq \frac{v_{i}^{2}}{2({k_{b_{i}}}^{2}-v_{i}^{2}{)}^{2}}+\frac{{\bar{\varepsilon }}_{i}^{2}}{2}$$

(30) $$\frac{v_{i}}{k_{b_{i}}^{2}-v_{i}^{2}}W_{i}^{*T}\left(\Phi_{i}(x)-\Phi_{i}(x_{i})\right)\leq \frac{\|W_{i}^{*}{\|}^{2}v_{i}^{2}}{(k_{b_{i}}^{2}-v_{i}^{2}{)}^{2}}+N$$

Substitute (29) and (30) into (28):

(31) D0CtqVi≤D0CtqVi−1+vikbi2−vi2(vi+1+ri+1+xi+1,d−zi+zi+W^iTΦi(xi)+W~iTΦi(xi)+di(t)−Dtq0Cxi,d−D0Ctqri+vi22(kbi2−vi2)2+‖Wi*‖2vi2(kbi2−vi2)2−1ΓiW~iTD0CtqWi−1γiΛ~iD0CtqΛi+N+ϵ¯i22

The pseudo control law $z_{i}$ and compensated signal $r_{i}$ are shown below:

(32)  zi=−ciSi−kbi2−vi2kbi−12−vi−12Si−1−Si2(kbi2−vi2)−Λitanhviϖ−W^iTΦi(xi)+D0Ctqxi,dD0Ctqri=−ciri−kbi2−vi2kbi−12−vi−12ri−1−ri2(kbi2−vi2)+ri+1+(xi+1,d−zi)

The corresponding adaptive law is as follows:

(33) D0CtqΛi=γivikbi2−vi2tanhviϖ−τiΛiD0CtqWi=Γivikbi2−vi2Φi(xi)−σiWi

Substitute (32) and (33) into (31):

(34)  D0CtqVi≤vivi+1kbi2−vi2+∑k=1i1kbk2−vk2−ck−‖Wk*‖2vk2+τkγkΛ~kΛk+σkΓkW~kTWk+Mk

where $M_{k}=kN+\sum_{k=1}^{i}0.2785\Lambda_{k}^{*}\varpi /(k_{b_{k}}^{2}-v_{k}^{2})+{\bar{\epsilon }}_{k}^{2}/2$.

**Step 4:** With a similar method mentioned above, the fractional order derivative of the compensated tracking error signal $v_{4}$ is calculated to be:

(35) D0Ctqv4=D0CtqS4−D0Ctqr4=−ω3ut+W4*TΦ4x+W~4TΦ4x4−W4*TΦ4x4+W^4TΦ4x4+ε4x+d4t−D0Ctqx4,d−D0Ctqr4

Choose the Lyapunov function as:

(36) $$V_{4}=V_{3}+\frac{1}{2}\ln\frac{k_{b_{4}}^{2}}{k_{b_{4}}^{2}-v_{4}^{2}}+\frac{1}{2\Gamma_{4}}{\tilde{W}}_{4}^{T}{\tilde{W}}_{4}+\frac{1}{2\gamma_{4}}{\tilde{\Lambda }}_{4}^{2}$$

According to Lemmas 1–3:

(37) D0CtqV4≤D0CtqV3+v4kb42−v42(−ω3u(t)+W4*TΦ4(x)+W~4TΦ4(x4)−W4*TΦ4(x4) +W^4TΦ4(x4)+ε4(x)+d4(t)−D0Ctqx4,d−D0Ctqr4)−1Γ4W~4TD0CtqW4−1γ4Λ~4D0CtqΛ4

According to Young’s inequality:

(38) $$\frac{v_{4}}{k_{b_{4}}^{2}-v_{4}^{2}}\varepsilon_{4}(x)\leq \frac{v_{4}^{2}}{2(k_{b_{4}}^{2}-v_{4}^{2}{)}^{2}}+\frac{{\bar{\varepsilon }}_{4}^{2}}{2}$$

(39) $$\frac{v_{4}}{k_{b_{4}}^{2}-v_{4}^{2}}W_{4}^{*T}\left(\Phi_{4}(x)-\Phi_{4}(x_{4})\right)\leq \frac{\|W_{4}^{*}{\|}^{2}v_{4}^{2}}{(k_{b_{4}}^{2}-v_{4}^{2}{)}^{2}}+N$$

Substitute (38) and (39) into (37):

(40)  D0CtqV4≤D0CtqV3+v4kb42−v42−ω3u(t)+W^4TΦ4(x4)+W4T~Φ4(x4)+d4(t)−D0Ctqx4,d−D0Ctqr4)+v422(kb42−v42)2+‖W4*‖2v42(kb42−v42)2−1Γ4W~4TD0CtqW4−1γ4Λ~4D0CtqΛ4+N+ε¯422

Design the real control law u(t) and compensated signal $r_{4}$ as follows:

(41) u(t)=−1ω3−c4S4−kb42−v42kb32−v32S3−S42(kb42−v42)−Λ4tanhv4ϖ−W^4TΦ4(x4)+D0Ctqx4,dD0Ctqr4=−c4r4−kb42−v42kb32−v32r3−r42(kb42−v42)

and the adaptive law is in the following form:

(42) D0CtqΛ4=γ4v4kb42−v42tanhv4ϖ−τ4Λ4D0CtqW4=Γ4v4kb42−v42Φ4(x4)−σ4W4

Substitute (41) and (42) into (40):

(43) D0CtqV4≤Mi+∑i=141kbi2−vi2−ci−‖Wi*‖2vi2+τiγiΛ~iΛi+σiΓiW~iTWi,

and $M_{i}=4N+\sum_{i=1}^{4}0.2785\Lambda_{i}^{*}\varpi /(k_{b_{i}}^{2}-v_{i}^{2})$.

**Figure 3:**
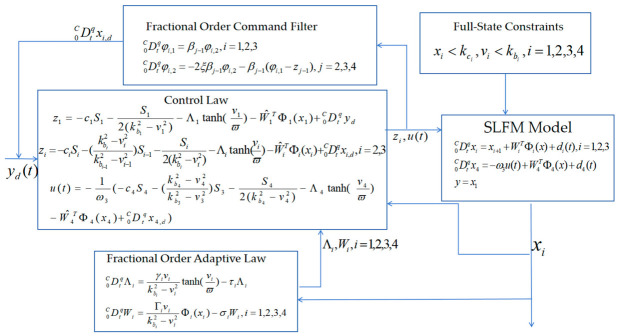
Block diagram for the controller.

The block diagram for the adaptive backstepping control algorithm based on the command filter and full-state constraints is shown in Figure 3:

## 4. Stability Analysis

After the establishment of the controller design, the proof that the variables occurred above are bounded is carried out in the proceeding chapter. For the sake of simplifying the stability analysis process, rewrite (43) in the following form:

(44)  D0CtqV4≤−∑i=14(ci−‖Wi*‖2)vi2kbi2−vi2+∑i=14τiγiΛ~iΛi+∑i=14σiΓiW~iTWi+ ∑i=14ϵ¯i22+∑l=140.2785Λi*ϖkbi2−vi2+4N#

It is already specified in the previous contents that $|\nu_{i}|<k_{b_{i}},\;\varpi >0,$ and the disturbance upper bound $\Lambda_{i}^{*}>0.$ Therefore, (44) can further be written as:

(45) D0CtqV4≤−∑i=14ci−‖Wi*‖2vi2kbi2−vi2+∑i=14τiγiΛ~iΛi+∑i=14σiΓiW~iTWi+∑i=14ϵ¯i22

Based on Young’s inequality and ${\tilde{W}}_{i}=W_{i}^{*}-W_{i}$, ${\tilde{\Lambda }}_{i}=\Lambda_{i}^{*}-\Lambda_{i}$ mentioned above, the following inequalities can be obtained:

(46) $$\frac{\tau_{i}}{\gamma_{i}}{\tilde{\Lambda }}_{i}\Lambda_{i}=-\frac{\tau_{i}}{\gamma_{i}}{\tilde{\Lambda }}_{i}^{2}+\frac{\tau_{i}}{\gamma_{i}}{\tilde{\Lambda }}_{i}\Lambda_{i}^{*}\leq -\frac{\tau_{i}}{2\gamma_{i}}{\tilde{\Lambda }}_{i}^{2}+\frac{\tau_{i}}{2\gamma_{i}}\Lambda_{i}^{*2}\#$$

(47) $$\;\frac{\sigma_{i}}{\Gamma_{i}}{\tilde{W}}_{i}^{T}W_{i}=-\frac{\sigma_{i}}{\Gamma_{i}}{\tilde{W}}_{i}^{T}{\tilde{W}}_{i}+\frac{\sigma_{i}}{\Gamma_{i}}{\tilde{W}}_{i}^{T}W_{i}^{*}\leq -\frac{\sigma_{i}}{2\Gamma_{i}}{\tilde{W}}_{i}^{T}{\tilde{W}}_{i}+\frac{\sigma_{i}}{2\Gamma_{i}}\|W_{i}^{*}{\|}^{2}$$

Meanwhile, the following inequality can be obtained according to Lemma 3:

(48) $$-\frac{\left(c_{i}-{\left\|W_{i}^{*}\right\|}^{2}\right)v_{i}^{2}}{k_{b_{i}}^{2}-v_{i}^{2}}\leq -\left(c_{i}-{\left\|W_{i}^{*}\right\|}^{2}\right)\ln\frac{k_{b_{i}}^{2}}{k_{b_{i}}^{2}-v_{i}^{2}}$$

Based on (46)–(48), rewrite (45) into the form below:

(49) D0CtqV4≤−∑i=14ci−Wi*2lnkbi2kbi2−vi2−∑i=14τi2γiΛ~i2−∑i=14σi2ΓiW~iTW~i+∑i=14τi2γiΛi*2+∑i=14σi2Γi‖Wi*‖2+∑i=14ϵ¯i22

Simultaneously, according to (18), (27) and (36), the equation below can be obtained:

(50) $$V_{4}=\frac{1}{2}\sum_{i=1}^{4}\ln\frac{k_{b_{i}}^{2}}{k_{b_{i}}^{2}-v_{i}^{2}}+\sum_{i=1}^{4}\frac{1}{2\Gamma_{i}}{\tilde{W}}_{i}^{T}{\tilde{W}}_{i}+\sum_{i=1}^{4}\frac{1}{2\gamma_{i}}{\tilde{\Lambda }}_{i}^{2}$$

Based on (49) and (50), obtain:

(51) D0CtqV4≤−ϑV4+M¯,

where $\vartheta =\min\left\{c_{i}-\|W_{i}^{*}{\|}^{2},\tau_{i}/2\gamma_{i},\sigma_{i}/2\Gamma_{i},i\;=\;1,\;2,\;3,\;4\right\}$ and $\bar{M}=\sum_{i=1}^{4}\tau_{i}\Lambda_{i}^{*2}/2\gamma_{i}+{\bar{\epsilon }}_{i}^{2}/2$+ $\sigma_{i}\|W_{i}^{*}{\|}^{2}/2\Gamma_{i}$.

Based on (51) and Lemma 4, it can be obtained that:

(52) $$V_{4}(t)\leq \left(V_{4}(0)-\frac{M}{\vartheta }\right)\phi_{q}(-\vartheta t^{q})+\frac{\bar{M}}{\vartheta },$$

where t≥0, M, ϑ>0, and $\bar{M}$ is bounded. Further rewrite (52) as:

(53) $$V_{4}(t)\leq V_{4}(0)\phi_{q}(-\vartheta t^{q})+\frac{\bar{M}}{\vartheta },\;t\geq 0$$

(54) $$\underset{t\to \infty }{\Rightarrow \lim}V_{4}(t)\leq \frac{\bar{M}}{\vartheta }$$

It can be derived that compensated tracking error signal $v_{i}$ is bounded and restricted in the compact set $\Omega_{v}$, where

(55) $$\Omega_{v}=\left\{v_{i}\;|\;|v_{i}|\leq k_{b_{i}}\sqrt{1-e^{-2V_{4}(0)\phi_{q}(-{\vartheta t}^{q})-2\frac{\bar{M}}{\vartheta }}},\;i\;=\;1,\;2,\;3,\;4\right\}$$

According to (51), (54) and (55), it can be obtained that $\ln\left(\frac{k_{b_{i}}^{2}}{k_{b_{i}}^{2}-v_{i}^{2}}\right)$ is bounded, which demonstrates that $\left|v_{i}\right|<k_{b_{i}}$. It can also be derived that ${\tilde{W}}_{i}$ and ${\tilde{\Lambda }}_{i}$ are bounded. Since ${\tilde{W}}_{i}=W_{i}^{*}-W_{i}$ and ${\tilde{\Lambda }}_{i}=\Lambda_{i}^{*}-\Lambda_{i}$, both $W_{i}$ and $\Lambda_{i}$ are bounded.

Next, prove the boundedness of tracking error $S_{i}$. As $S_{i}=v_{i}+r_{i}$, the first step is to prove that compensated signal $r_{i}$ is bounded. According to [21], one has that the error of command filter satisfies $\|g_{i}(x_{i+1,d}-z_{i})\|\leq \mu \rho ,i\;=\;1,\;2,\;3$. $g_{i}=1$ is the gain coefficient of the command filter error system. The upper bound is ρ, and μ is the error’s upper bound of the command filter.

Use the following Lyapunov function:

(56) $$V_{r}=\sum_{i=1}^{4}\frac{1}{2}r_{i}^{2}$$

The fractional order derivatives of all compensated signals are as follows:

(57)  D0Ctqr1=−c1r1−r12(kb12−v12)+r2+(x2,d−z1)D0Ctqri=−ciri−kbi2−vi2kbi−12−vi−12ri−1−ri2(kbi2−vi2)+ri+1+(xi+1,d−zi)D0Ctqr4=−c4r4−kb42−v42kb32−v32r3−r42(kb42−v42),i = 2, 3

The fractional order derivative of $V_{r}$ is:

(58)  D0CtqVr≤−∑i=14ci+12(kbi2−vi2)ri2+∑i=241−kbi2−vi2kbi−12−vi−12ri−1ri+∑i=24ri−1xi,d−zi−1#

Based on Young’s inequality, the eventual form of $V_{r}$ is:

(59) D0CtqVr≤−c1−12kb12−v12+121−kb22−v22kb12−v12r12+−c2−12kb22−v22+121−kb22−v22kb12−v12+121−kb32−v32kb22−v22r22+−c3−12kb32−v32+121−kb32−v32kb22−v22+121−kb42−v42kb32−v32r32+−c4−12kb42−v42+121−kb42−v42kb32−v32r42+μ22∑i=13ri2≤−ψVr+m0μ2ρ2

By selecting the appropriate ψ, the inequality below can be satisfied:

(60) $$\underset{t\to \infty }{\lim}V_{r}(t)\leq \frac{m_{0}\mu^{2}\rho^{2}}{\psi }$$

As a result, $r_{i}$ is bounded, and this means $S_{i}$ is bounded. Meanwhile, the pseudo control law $z_{1}$ is a function of $S_{1},\;k_{b_{1}},v_{1},\Lambda_{1},W_{1}$ and D0Ctqyd, so it is derived that $z_{1}$ is continuous and bounded. Similarly, it can be obtained that pseudo control laws $z_{2},\;z_{3}$ and the real control law u(t) are all bounded.

For the command filter:

(61) D0Ctqφi,2=−2ξβj−1φi,2−βj−1φi,1−zj−1≤−2ξβj−1φi,2+βj−1μ

Therefore, both D0Ctqφi,2 and $\varphi_{i,2}$ are bounded, and the boundedness of D0Ctqφi,1 can be acquired by D0Ctqφi,1=βj−1φi,2i,j = 2, 3, 4. From Assumptions 5 and 6, under the initial conditions that $\Omega_{x_{1}}=\{x_{i}(0)\in R:|x_{i}(0)|<k_{c_{i}},k_{i}>0\}$, all the pseudo control laws, adaptive laws, and compensated signals specified in Section 3 are effectively designed, making sure that all the signals in the system are bounded. Additionally, as $v_{i}=x_{i}-x_{i,d}-r_{i}$ and $x_{i,d}<k_{a_{i}}(t)$, it can be obtained that $|x_{i}|<k_{b_{i}}(t)+k_{a_{i}}(t)+r_{i}$. Define $k_{b_{i}}(t)=k_{c_{i}}(t)-k_{a_{i}}(t)-r_{i}$, and then it is clear that $\left|x_{i}|<k_{c_{i}}(t\right)$. To conclude, all the states in the closed-loop system satisfy the time-variant state constraints specified previously.

## 5. Simulation Results and Discussions

For the controller designed above, the simulation results obtained by Matlab 2022b are shown in this chapter, and the selection of parameters is specified in Table 1 below.

**Figure 4:**
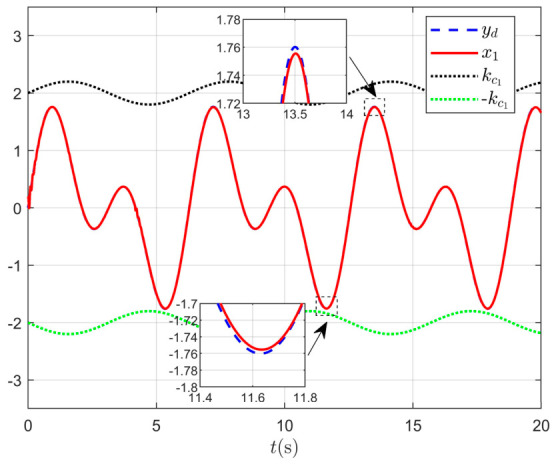
Tracking performance and $k_{c_{1}}(t).$

For the neural network, the parameters are given as: μ1 = [−1 −0.5 0 0.5 1;−1 −0.5 0 0.5 1;−1 −0.5 0 0.5 1;−1 −0.5 0 0.5 1;−1 −0.5 0 0.5 1;−1 −0.5 0 0.5 1;−1 −0.5 0 0.5 1;−1 −0.5 0 0.5 1]; μ2 = [−1 −0.5 0 0.5 1;−1 −0.5 0 0.5 1;−1 −0.5 0 0.5 1;−1 −0.5 0 0.5 1;−1 −0.5 0 0.5 1;−1 −0.5 0 0.5 1;−1 −0.5 0 0.5 1;−1 −0.5 0 0.5 1]; μ3 = [−1 −0.5 0 0.5 1;−1 −0.5 0 0.5 1;−1 −0.5 0 0.5 1;−1 −0.5 0 0.5 1;−1 −0.5 0 0.5 1;−1 −0.5 0 0.5 1;−1 −0.5 0 0.5 1;−1 −0.5 0 0.5 1]; μ4 = [−1 −0.5 0 0.5 1;−1 −0.5 0 0.5 1;−1 −0.5 0 0.5 1;−1 −0.5 0 0.5 1;−1 −0.5 0 0.5 1;−1 −0.5 0 0.5 1;−1 −0.5 0 0.5 1;−1 −0.5 0 0.5 1]; X1 = [x1;x2;x3;x4;v1;v2;v3;v4]; X2 = [x1;x2;x3;x4;v1;v2;v3;v4]; X3 = [x1;x2;x3;x4;v1;v2;v3;v4]; and X4 = [x1;x2;x3;x4;v1;v2;v3;v4]. The function is designed as: $\phi_{i}\left(x\right)=\exp\left(X_{i}-\mu_{i}\left(:,j\right)\right)\left(j=1,2,3,4,5;i=1,2,3,4\right)$. The initial conditions for other parameters are 0. The tracking performance of SLFM, indicated by how well the output $y=x_{1}$ tracks the reference signal $y_{d}$, and the time responses for the time-varying constraints $-k_{c_{1}}(t)$ and $k_{c_{1}}(t)$ for $x_{1}$ are shown in Figure 4. $x_{1}$ is the sum of rotation angle θ of the manipulator and the bending deformation α at the tip.

**Figure 5:**
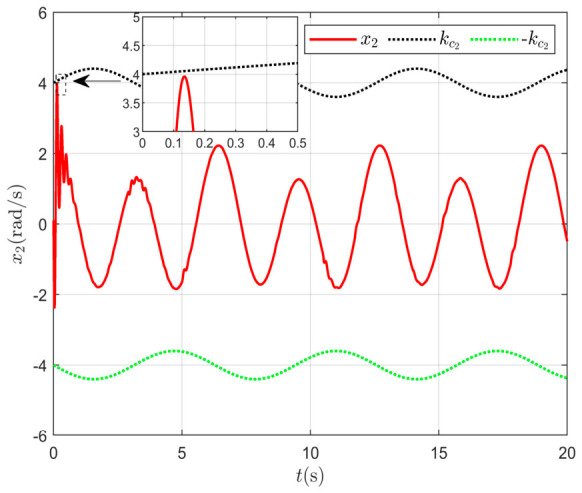
Tracks for $x_{2}$ and $k_{c_{2}}(t).$

The track for $x_{2}$ and time responses for time-varying constraints −$k_{c_{2}}(t)$ and $k_{c_{2}}(t)$ for $x_{2}$ are shown in Figure 5. $x_{2}$ is the rate of change for $x_{1}$.

**Figure 6:**
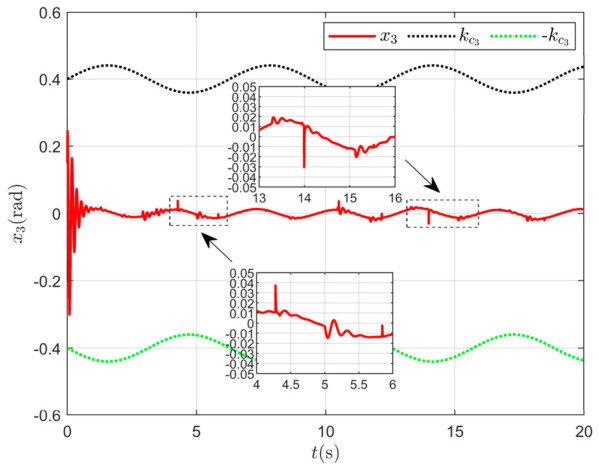
Tracks for $x_{3}$ and $k_{c_{3}}(t)$.

The track for $x_{3}$ and time responses for time-varying constraints −$k_{c_{3}}(t)$ and $k_{c_{3}}(t)$ for $x_{3}$ are shown in Figure 6. $x_{3}$ is bending deformation α at the manipulator’s tip.

**Figure 7:**
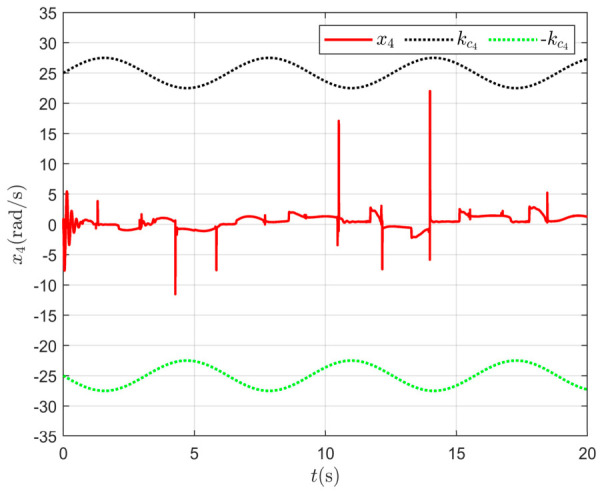
Tracks for $x_{4}$ and $W_{4}.$ and $k_{c_{4}}(t).$

The track for $x_{4}$ and time responses for time-varying constraints −$k_{c_{4}}(t)$ and $k_{c_{4}}(t)$ for $x_{4}$ are shown in Figure 7. $x_{4}$ is the rate of change for $x_{3}$.

**Figure 8:**
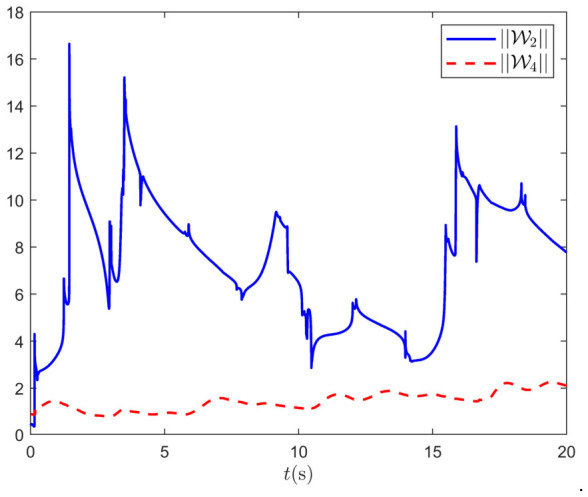
Tracks of adaptive parameters $W_{2}$ and $W_{4}$.

The time responses of adaptive parameters $W_{2}$ and $W_{4}$ are shown in Figure 8.

**Figure 9:**
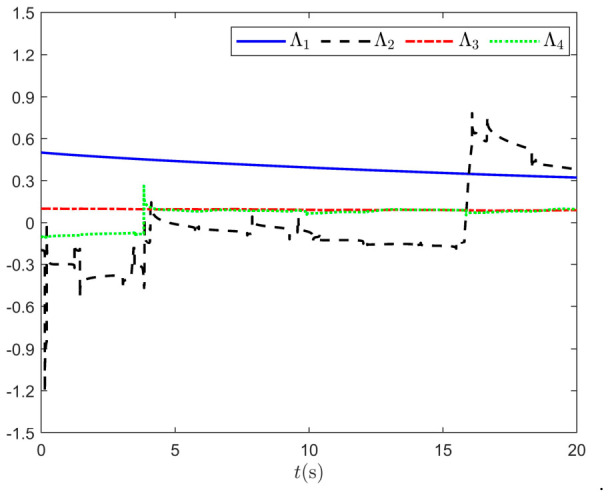
Tracks for adaptive parameters $\Lambda_{i},\;i=1,\;2,\;3,\;4.$

The time responses for adaptive parameters $\Lambda_{i},\;i=1,\;2,\;3,\;4$ are shown in Figure 9.

**Figure 10:**
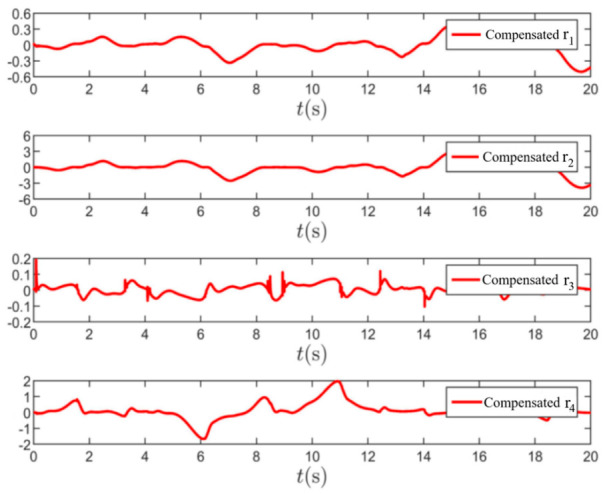
Tracks for compensated signals $r_{i},\;i=1,\;2,\;3,\;4.$

The time responses for compensated signals $r_{i},\;i=1,\;2,\;3,\;4$ are shown in Figure 10.

**Figure 11:**
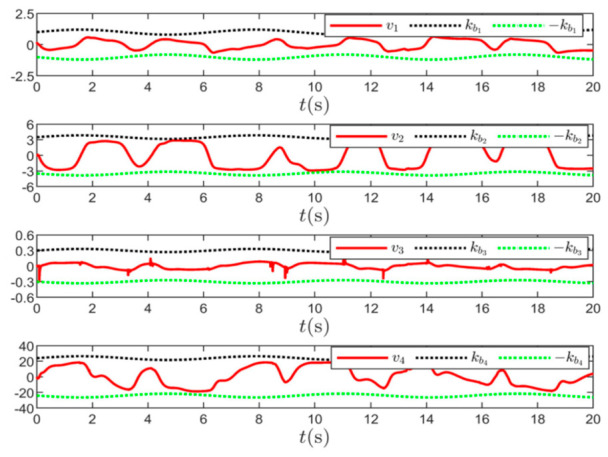
Tracks for compensated tracking error signal $v_{i}$ and constraint $k_{b_{i}}$, *i* = 1, 2, 3, 4. The time response of the system’s control input signal *u*(*t*) is shown in Figure 12.

The time responses for compensated tracking error signals $v_{i},\;i=1,\;2,\;3,\;4$ and corresponding constraints $k_{b_{i}}$ are shown in Figure 11.

**Figure 12:**
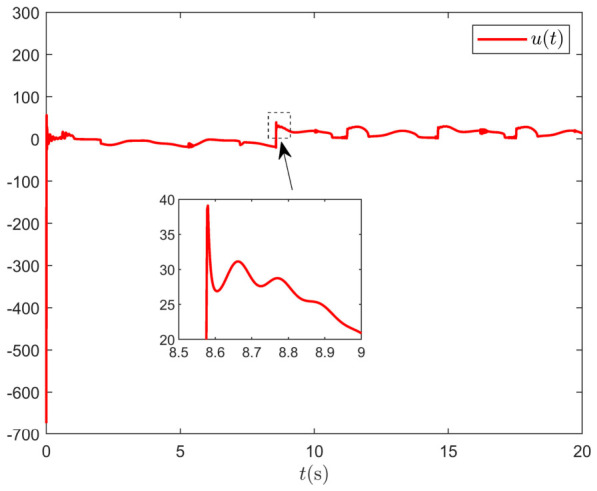
Track for control input *u*(*t*).

From Figure 4, Figure 5, Figure 6 and Figure 7, it can be seen that all the state variables in the system are strictly constrained in the predefined time-varying intervals, and the output signal $x_{1}$ can effectively track the reference signal $y_{d}$, with the tracking error being no more than 0.02 rad. This directly indicates the effectiveness and the satisfactory performance of the controller proposed in this article.

It is worth noting that Figure 6 shows the track of the tip deflection angle of the SLFM. When the SLFM’s link arrives at the desired angle, the elastic vibration of the system does not converge to 0, which will bring out a lagging effect for SLFM’s angle tracking and influence the overall performance. It can be seen from Figure 6 that the controller designed in this article suppresses the tip deflection angle to be less than 0.045 rad, dramatically decreasing the negative effects of the tip’s vibration.

**Figure 13:**
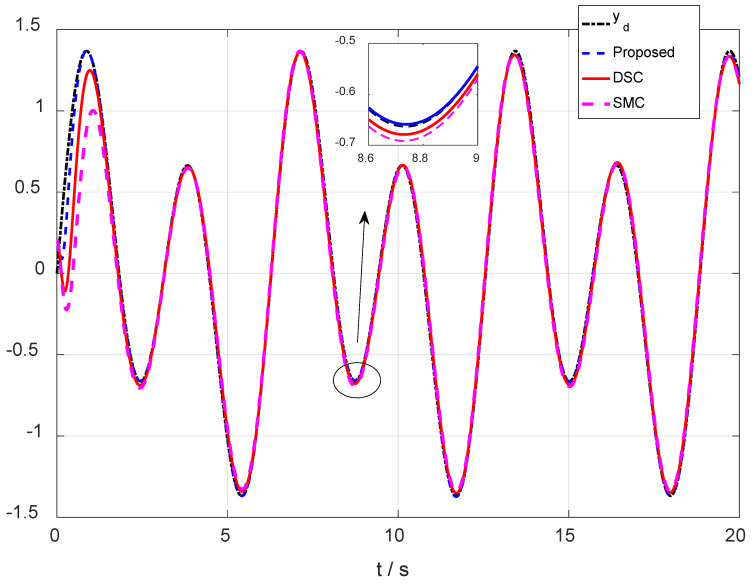
Rotational angle tracking performance comparison.

**Figure 14:**
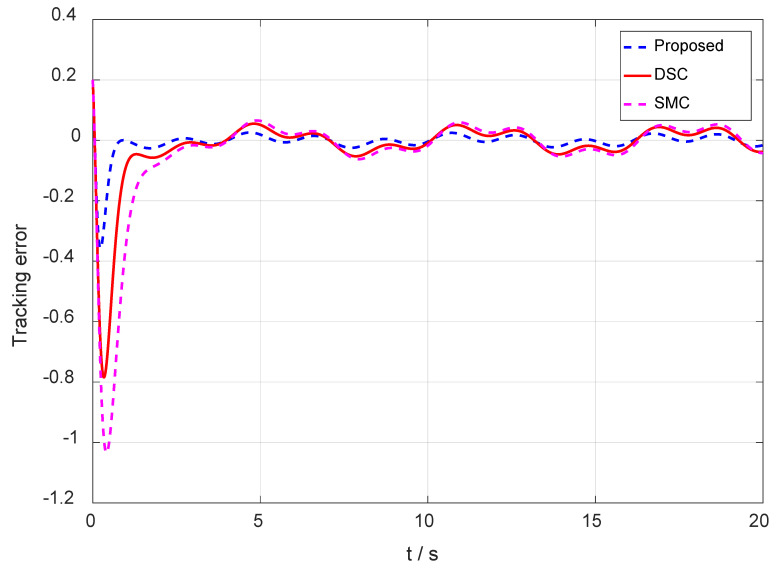
Tracking error ($S_{1}$) comparison.

Finally, in order to validate the superiority of the algorithm proposed in this article in terms of the tracking performance, make a comparison with the dynamic surface control method (DSC) and the sliding mode control (SMC). Take the reference signal to be $y_{d}=sin(2t)+0.5sin(t)$, and set the parameters of the DSC controller to be $c_{1}=150,\;c_{2}=15,\;c_{3}=20,\;c_{4}=40$. Set the parameters of the controller proposed in this article to be $c_{1}=600,\;c_{2}=15,\;c_{3}=20,\;c_{4}=20$, and the sliding mode control method is designed as S=ce. The other parameters are kept the same. The comparisons between SLFM’s rotational angle tracking performance and tracking error ($S_{1}$) are shown in Figure 13 and Figure 14, respectively. It can be concluded that the method proposed in this article has better tracking performance with a smaller tracking error than DSC and SMC. The Root Mean Square Errors (RMSEs) of the tracking errors with different methods are shown in Table 2, from which one can see that the tracking performance of the proposed method is better than the other two methods.

Future work may be done in fields like establishing a physical SLFM experiment platform to further validate the effectiveness of the proposed method. Meanwhile, a more precise model of SLFM may be derived to better capture the dynamic behavior of SLFM and yield a more satisfactory control performance.

## 6. Conclusions

This paper investigates the trajectory tracking control of a fractional-order single-link flexible manipulator (SLFM) system under symmetric time-varying full-state constraints. The symmetric time-varying barrier Lyapunov function (BLF) is incorporated into each step of the backstepping design to ensure that all states remain within the prescribed bounds, thereby enhancing system safety. An adaptive RBF neural network controller with command filters is developed to handle uncertainties and avoid the complexity explosion issue. Lyapunov theory proves that all closed-loop signals are semi-globally uniformly ultimately bounded, and simulations verify the effectiveness of the proposed method. The simulation results show that the presented controller has excellent control performance, the tracking error is less than 0.02 rad, and the tip polarization angle of the system does not exceed 0.045 rad. Additionally, the comparison with the recent DSC and SMC methods shows that the designed controller behaves with less tracking error and has a better control performance, which in return validate the effectiveness and superiority of the proposed control strategy. Future work will focus on experimental validation via a physical flexible manipulator platform and on developing more refined models to better capture system dynamics and improve control performance.

## Figures and Tables

**Table 1:** Parameters selection.

Category	Parameters
Dynamic model parameters	${\;\;B}_{eq}=0.6153$ Nm, $J_{eq}=0.0156\;kg\cdot m^{2},$ $J_{l}=0.0312\;kg\cdot m^{2},$ $K_{s}=7.7886\;N\cdot m/rad$
Controller parameters	$c_{1}=400,\;c_{2}=15,\;c_{3}=20$ and $c_{4}=30$
Command filter parameters	$\xi =0.8,\;\beta_{1}=30,\;\beta_{2}=30$ and $\beta_{3}=6$
Adaptive parameters	$\sigma_{1}=\sigma_{2}=\sigma_{3}=\sigma_{4}=0.1,\;\Gamma_{1}=\Gamma_{2}=\Gamma_{3}=\Gamma_{4}=30.2,$ $\gamma_{1}=\gamma_{2}=\gamma_{3}=\gamma_{4}=0.1,$ $\tau_{1}=\tau_{2}=\tau_{3}=\tau_{4}=0.03$
Gaussian function width	$\eta_{1}=\eta_{2}=\eta_{3}=\eta_{4}=1.5$
RBF NN nodes	$\iota_{1}=\iota_{2}=\iota_{3}=\iota_{4}=5$
State constraints	$k_{c_{1}}=2+0.2\sin(t),\;$ $k_{c_{2}}=4+0.4\sin(t),$ $k_{c3}=0.4+0.04\sin(t),\;k_{c4}=25+2.5\sin(t)$
Initial conditions of system	$x_{1}(0)=0.2,x_{2}(0)=0.1,x_{3}\left(0\right)=0.15,x_{4}(0)=0.05$
Initial adaptive parameters	$\Lambda_{1}(0)=0.5,\Lambda_{2}(0)=-0.2,\Lambda_{3}(0)=0.1,\Lambda_{4}(0)=-0.1$
Initial NN weights	$w_{1}(0)=[0.1,\ldots ,0.1{]}_{1\times 5}^{T},w_{2}(0)=[0.2,\ldots ,0.2{]}_{1\times 5}^{T},$ $w_{3}(0)=[0.3,\ldots ,0.3{]}_{1\times 5}^{T},w_{4}(0)=[0.4,\ldots ,0.4{]}_{1\times 5}^{T}$
Unknown disturbances	$d_{1}(t)=0.05\sin(\pi t),d_{2}(t)=0.03\cos(\pi t),$ $d_{3}(t)=0.02\sin(t),\;d_{4}(t)=0.01\cos(t)$
System order	q=0.9
Reference signal	$y_{d}=sin(t)+sin(2t)$
Simulation time	20s

**Table 2:** Comparison results of different methods.

Method	Proposed	DSC	SMC
RMSE	0.041944	0.11158	0.16959
Max error	0.35854	0.78436	1.0343

## Data Availability

The original contributions presented in this study are included in the article. Further inquiries can be directed to the corresponding author.
